# How Intraday Index Changes Influence Periodontal Assessment: A Preliminary Study

**DOI:** 10.1155/2017/7912158

**Published:** 2017-07-30

**Authors:** Carlo Bertoldi, Andrea Forabosco, Michele Lalla, Luigi Generali, Davide Zaffe, Pierpaolo Cortellini

**Affiliations:** ^1^Department of Surgery, Medicine, Dentistry and Morphological Sciences with Transplant Surgery, Oncology and Regenerative Medicine Relevance, University of Modena and Reggio Emilia, Modena, Italy; ^2^Department of Economics Marco Biagi, University of Modena and Reggio Emilia, Modena, Italy; ^3^Department of Biomedical, Metabolic and Neural Sciences, University of Modena and Reggio Emilia, Modena, Italy; ^4^European Research Group on Periodontology (ERGO Perio), Bern, Switzerland

## Abstract

It is reputed that periodontal indices remain unchanged over a 24-hour period, with great clinical significance. This preliminary study analyzes daily index changes. In 56 selected patients, full-mouth plaque score (FMPS), full-mouth bleeding score (FMBS), periodontal screening and recording (PSR) indices, and periodontal risk assessment (PRA) were recorded at baseline and three times per day (check-I: 08.30, check-II: 11.30, and check-III: 14.30), after appropriate cause-related therapy. Correlation between variables was statistically analyzed by Stata. All periodontal indices improved at the examination phase. Statistical differences were detected for FMPS comparing all thrice daily checks. Statistical differences were detected for FMBS and PRA comparing check-III with check-I and check-II. PSR showed no significant changes. The worst baseline indices produced the widest daily fluctuation at the examination phase. Significant variation of indices is directly related to clinical severity of periodontal conditions at baseline. Patients affected by severe periodontal disease may show significantly greater index changes. As indices are routinely recorded only once per day, the index daily variation has clinical significance. This greatly affects therapeutic strategy as correct periodontal assessment requires multiple evaluations at standardized times, particularly when baseline conditions are severe.

## 1. Introduction

Maintenance of integrity and of biological rehabilitation, function, and aesthetics are principal objectives in general dentistry and periodontology [1, 2]. Dental health can be jeopardized to varying degrees by different periodontal diseases [3–7]. Periodontal diseases are oral disorders, characterized by gingival and periodontal inflammation, attachment loss, and alveolar bone resorption [4, 6, 7]. Oral microbiota, immune and inflammatory mediation, gene regulation, and hormonal changes play important roles in the onset of periodontal disease [8–10]. However, several additional causes, sometimes linked to systemic diseases, but also linked to psychological aspects or lifestyle, play roles in the progression of periodontal damage. Wide evidence exists in that daily stressors and stress vulnerability factors are associated with inflammatory markers and endocrine and immune functioning [5, 10, 11]. Moreover, it is known, thanks to pioneering work on animals, that response to various pathogens and their by-products including bacterial endotoxins and exotoxins and proinflammatory cytokines is under diurnal control [12–19]. Thaiss et al. [19] report that intestinal microbiota undergoes diurnal oscillation, controlled by host feeding time. Moreover, short-term rhythmic oscillations in intestinal microbiota may be exaggerated or disrupted under various disease conditions, impacting progression of microbiota-mediated diseases with different manifestations or with varying degrees of severity at different times of day [19–21]. The mucosal immune system found inside the mouth is similar to that of the small intestine [22, 23]. Dendritic cells, lymphocytes, and mucosal-associated lymphoid tissue (in the tonsils and lymphoid follicles) help to sample contents entering the mouth and determine “friend or foe.” Resident bacteria in the oral cavity are critical to this process. However, outside of dental circles, little attention has been paid to this fact until now [24] or to the 45% overlap of microbes found both in the mouth and in the colon [25]. Sato et al. [26], after studying the oral bacteriome, observed that some bacterial species exhibit significant changes in abundance over time while the majority of species exhibited no periodic changes over the course of a day. However, inter- and intraindividual variability and stability of the human microbiome remain poorly characterized, particularly at the intraday level [26].

Moreover, prevalence of periodontitis in several countries is based upon indices of periodontal treatment screening [27–29]. Consequently, clinical periodontal indices assume particular epidemiological, diagnostic, and prognostic importance in risk assessment and identification of appropriate therapeutic strategy, given the lack of reliable pathogenic criteria based specifically upon interpretation of type of inflammation [4, 5, 30, 31]. Probing depth and clinical attachment levels are used in the diagnosis or prognosis of specific periodontal diseases, but these indicators are liable to misinterpretation, even when correctly measured and even when abundant microbiota deposition or gingivitis is present [32–34]. These situations are frequent mainly during early stages of periodontal therapy. Conservative, orthodontic, and prosthetic issues, individual sensitivity to risk factors, lifestyle, and pathological state requiring treatment, motivate patients and achieve as standardized a periodontal condition as possible. Cause-related periodontal therapy is always performed during the oral hygiene education phase to fulfill this purpose. The cause-related treatment reduces periodontal inflammation and contributes to greater patient status standardization [35].

The community periodontal index of treatment needs (CPITN) and the periodontal screening and recording (PSR) score are indices recommended by both the American Dental Association and the American Academy of Periodontology as screening tools to facilitate early detection and periodontal disease treatment requirements [6, 36]. These indices are not, however, a substitute for periodontal charting during clinical and therapeutic periodontal evaluation but have great importance during the initial stages of patient evaluation. The full-mouth plaque score (FMPS) evidences presence of microbiota [37] while full-mouth bleeding score (FMBS) [38] evidences periodontal inflammation.

Periodontal risk assessment (PRA) consists in a functional diagram that helps the clinician to determine the risk of disease progression in individual patients [39] and is particularly effective in compliant subjects not suffering from aggressive periodontal disease [32, 40].

Treatment needs and management of periodontal disease depend largely upon accurate and reliable index recording, development of an appropriate treatment plan, and subsequent monitoring. However, since pioneering work by Hoover and Lefkowitz [41], gingival inflammation has been considered as a fluctuating disease with a daily cycle of change.

At present, periodontal indices are routinely recorded presupposing that they should not vary significantly, irrespective of the time of day. The aim of this study is to evaluate periodontal FMBS, FMPS, PSR, and PRA indices intraday changes during supportive periodontal therapy.

## 2. Materials and Methods

### 2.1. Study Population

The present study population was selected from a list of systemically healthy subjects referred for periodontal treatment, examined, and treated with cause-related therapy.

Checks or improvement studies in health care without external funding, performed by a clinician group working in a single sanitary structure, require no ethical committee approval according to Italian law (standard operating procedure of the provincial ethical committee of Modena, revision November 16, 2010).

All patients signed informed consent in which all procedures of the study were detailed. The research was conducted in full accordance with ethical principles, including the 2013 WMA Helsinki Declaration [42].

Subjects meeting the criteria reported in Table 1 were enrolled.

Enrolled subjects were affected by only mild periodontitis [43, 44] and had not undergone periodontal treatment during the previous year. Additionally, after completion of cause-related therapy, subjects had to be considered good compliers [1, 45, 46].

Each patient in this study had to meet all the above criteria in every phase of the clinical trial.

### 2.2. Study Design

Before patient examination and treatment, a pretrial calibration session was performed by two examiners on 10 healthy volunteer patients. Number of teeth present in the oral cavity (NoT), FMPS, FMBS, and PSR were recorded three times a day to obtain acceptable intra- and interexaminer clinical periodontal parameter assessment reproducibility.

The first step was the patient screening phase, anamnesis, preliminary dental visit, motivation, and preliminary treatment after patient consent. Gingival tissue sanitization was necessary to achieve a predictive periodontal diagnosis particularly in patients lacking dental supportive therapy or even with no history of regular dental examination for an extended period of time. This step concerned initial dental hygiene treatment and acute and urgent surgical, endodontic, and conservative conditions requiring a short-term solution to stabilize periodontal tissues.

The study opening visit was performed after completion of the first step of causal therapy. The opening visit (baseline, b) was carried out with a flat rhodium-plated dental mirror, dental probe, and periodontal probe with 1-millimeter marks (modified Click-Probe, Kerr Corp., Bioggio, Switzerland). Full-mouth plaque score at baseline (FMPS-b), by plaque disclosing gel detection, and full-mouth bleeding score at baseline (FMBS-b) were assessed at six sites per tooth. Periodontal screening and recording at baseline (PSR-b) was assessed using the World Health Organization (WHO) periodontal probe. PSR was measured for each tooth and sextant, but only the peak index value for each patient was considered. Finally, PRA-b was calculated.

The following variables were routinely recorded:

- age;
- gender;
- body mass index (BMI) as previously described [7];
- number of teeth (NoT) present in the oral cavity;
- glycemia (Gly).

Smoking habits (number of cigarettes per day [NoC]; current smoker, nicotinism; former smoker or nonsmoker, and, if smoker, the number of years as smoker) were recorded. Required cause-related therapy, including scaling and root planning, was completed and oral hygiene instructions were given. Indices at baseline were only considered in patients who were subsequently enrolled in the study.

### 2.3. Study Outcomes and Data Analysis

Five weeks after completion of the previous periodontal therapy, all patients were reevaluated. Patients fulfilling the study inclusion criteria and needing periodontal supportive therapy were enrolled and a new examination (examination phase) was performed the next week.

Each subject would be present at 3 different times (in a single day) at the periodontal examination and maintained usual daily routines. Checks (examination phase) were scheduled (check-I: 08.30, check-II: 11.30, and check-III: 14.30).

FMPS, FMBS, PSR, and PRA were considered during each check. NoT was considered once during examination phase. These data were compared with recorded clinical indices, gender, and smoking habits.

### 2.4. Statistical Analyses

Inter- and intrarate comparisons were carried out using Spearman coefficients for the number of teeth, PSR, FMBS, FMPS, and PRA.

Comparisons between initial and check-I, check-II, and check-III values for the considered variables were carried out using the Wilcoxon signed-rank statistic (matched data), while, for binary variables gender and smoking habit, comparisons between two independent groups (unmatched data) were carried out using the Mann–Whitney test. Spearman correlation coefficients between variables were calculated and the null hypotheses that no relationship exists between the pairs of variables were tested against the alternative (two-tailed) hypotheses that a relationship exists. FMPS, FMBS, PSR, and PRA were identified as dependent variables. The relationship between dependent and independent (covariates) variables was examined using the seemingly unrelated regression (SUR) model. SUR was used to analyze the effects of covariates on the level of the dependent variables on their maximum observed between the check at the examination phase equal to I, II, and III (that is, Δ-check, the maximum gap between check-I, check-II, and check-III). The level of significance of the applied tests was the standard value *α* = 0.05.

Multivariate analysis of covariance (MANCOVA) for repeated measurement was carried out for examination phases in order to profile index changes over time and to compare SUR results.

Statistical analysis was performed using Stata, version 14.00 (StataCorp LP, Lakeway Drive, College Station, TX, USA) [47].

## 3. Results

Spearman test analysis of the calibration session, performed by the two examiners on 10 healthy volunteer patients and concerning NoT, FMPS, FMBS, and PSR, produced intrarater agreement ranging between 0.782 and 1.000 (*p* < 0.001) and interrater agreement ranging between 0.771 and 1.000 (*p* < 0.001).

Sixty-seven patients met all requirements of the experimental protocol and study design. However, a total of 11 patients were excluded: 3 patients did not present at maintenance therapy visits conflicting with the study's stringent inclusion criteria; 6 patients (two females and four males) missed the examination phase; 1 patient moved to a distant city; and 1 patient became pregnant. Therefore, 28 females (16 nonsmokers and 12 smokers) and 28 males (17 nonsmokers and 11 smokers), aged 19–91 years (mean ± SD, 43.2 ± 15.7), formed the test population.

### 3.1. Indices at Baseline

Ranges in nonsmokers were NoT 10–31, FMPS-b 20–67.4%, FMBS-b 2.4–41.3%, PSR-b 1.0–2.5, and PRA-b 2.6–68.4. Ranges in smokers were NoT 10–30, FMPS-b 12.9–68.0%, FMBS-b 2.4–43.1%, PSR-b 0.83–2.0, and PRA-b 8.7–68.4. The greater FMPS-b and the lower PRA-b of nonsmokers were statistically significant in both genders when compared with smokers (Table 2).

### 3.2. Indices at Examination Phase

At check-I, ranges in nonsmokers were FMPS 6.5-38.5%, FMBS 0.7–25.0%, PSR 0.03–1.2, and PRA 2.6–46.7. At check-I, ranges in smokers were FMPS 7.0–35.3%, FMBS 1.2-19.2%, PSR 0.7–1.2, and PRA 8.7–29.9 (Table 2).

At check-II, ranges in nonsmokers were FMPS 3.3–35.0%; FMBS 0.5–25.0%; PSR 0.2-1.2; PRA 2.6–46.7. At check-II, ranges in smokers were FMPS 5.0–28.9%; FMBS 1.2–25.6%; PSR 0.8–1.2; PRA 7.8–34.6 (Table 2).

At check-III, ranges in nonsmokers were FMPS 3.3–28.4%, FMBS 0.01–22.8%, PSR 0.01–1.17, and PRA 2.60–43.30. At check-III, ranges in smokers were FMPS 5.6–36.0%, FMBS 2.0–18.5%, PSR 0.67–1.17, and PRA 8.66–25.12 (Table 2).

### 3.3. Clinical Outcomes

In all patients, FMPS, FMBS, PSR, and PRA (Table 2) highlighted statistically significant clinical improvements from baseline to the examination phase. In all patients, statistically significant differences were recorded between check-I, check-II, and check-III. PSR was almost not significant, whereas FMBS, FMPS, and PRA were often significant (Table 3).

Check-I and check-II FMPS values were significantly different in females, whereas check-I FMBS value was significantly different from check-II and check-III FMBS values in males (Table 3). Check-I, check-II and check-III PSR values were not statistically different in either females or males. Check-II and check-III PSR values were significantly greater in females than in males (Table 2). Check-I, check-II and check-III PRA values were statistically different in males, but not in females (Table 3).

Check-I FMPS value was significantly different from check-III FMPS value in nonsmokers (Table 3). Check-III FMBS values were significantly different from check-II FMBS value in nonsmokers and check-I FMBS value in smokers. Check-I PSR value was significantly different from check-II PSR value in smokers. Check-III PRA value was significantly different from check-I and check-II PRA values in nonsmokers, whereas check-I PRA value was significantly different from check-II and check-III PRA values in smokers (Table 3).

Several significant positive correlations between dental indices at examination phases (check-I, check-II, and check-III) were found after the Spearman test was performed (Table 4).

### 3.4. SUR Analysis

SUR multivariate analysis highlighted several statistically significant relationships between dental, individual, and lifestyle indices versus periodontal indices (Table 5).

Older patients showed a significant decrease of 1.3 units of FMPS-Δ and 0.5 units of FMBS-Δ relative to the expected indices of patients 10 years younger. Women showed a significantly decreased PSR-Δ. An increase of 20 cigarettes per day caused a significant PSR-Δ reduction of 0.4 units (Table 5), whereas smokers (nicotinism) showed an increased PRA-Δ of approximately 4 units.

The 20% increase in FMBS at baseline (FMBS-b) produced a significant increase of FMBS-Δ (Δ-check, the maximum gap between check-I, check-II, and check-III) of 3.2%, PRA-Δ of 1.6 units, and a minimal decrease of PSR-Δ. The 2-unit increase of PSR-b produced a significant increase of approximately 11% FMPS-Δ. The 20-unit increase of PRA-b produced a significant increase of PSR-Δ of 0.2 units (Table 5).

SUR analysis of baseline values is reported in Table 5.

## 4. Discussion

Periodontal screening tests require probing to assess periodontal attachment loss circumferential to each dental element or dental implant. To assess the risk of disease progression, a useful tool can be found in PRA (periodontal risk assessment), consisting of a functional diagram [39]. PSR is a useful test for periodontal screening as it is sensitive, specific, inexpensive, quick, and simple to perform, limiting the number of possible errors associated only with probing procedures [28, 29, 31, 33]. This present study was performed relating to the initial stages of periodontal examination and supportive treatment. PSR is particularly useful during the initial phase and is particularly important in order to determine periodontal treatment requirements [6, 36, 46]. FMBS and FMPS are, respectively, indices of inflammation and of microbiota presence and should be obtained early. Regarding systemic predisposition to inflammatory disorders, several risks and prognostic factors are probably challenges that trigger or worsen periodontal disorders. Therefore, patients affected by systemic and oral health conditions that could potentially introduce disturbing variables were excluded [5, 48, 49]. Stringent enrollment criteria were applied and the decision to perform the examination phase was made only after and temporally close to a positive response to causal therapy in order to achieve a reliable clinical outcome and an excellent standard of oral clinical settings, thus avoiding potentially perturbing variables.

Enrolled patients were affected by only mild periodontitis, but not by moderate or severe periodontitis [43, 44]. The nosology of periodontitis is complex and currently being developed and studied. In this present study, patients affected by advanced periodontitis were excluded. This decision was made to reduce the impact of major confounding variables resulting from the complexity of severe periodontitis which exacerbates other risk conditions and frequently entails elaborate and significantly different therapeutic strategies [2, 4, 43, 44, 50, 51].

Different forms of periodontal disease require different therapeutic strategies. Furthermore, a specific periodontal lesion may not always be treatable in the same manner, but rather on a case-specific basis [52, 53]. Further research, therefore, may suddenly present new therapeutic strategies [54, 55]. Consequently, it may be more difficult to obtain examiner agreement regarding patients with moderate or severe periodontitis due to haste and/or inaccurate, immoderate assessment [56–59].

The use of specific dental indices, specific periodontal probes, and a preventive calibration session make examiner agreement more obtainable and reliable [56, 59, 60]. Furthermore, patients progressing to the examination phase of this present study were required not to change their daily habits and were examined delicately three times in one day. A large number of exogenous variables potentially able to produce misleading results during the examination phase were eliminated thus creating the necessary conditions to obtain significantly similar measurements. Nevertheless, baseline data recorded using the same accurate method used during the three check phases were included in order to detect possible influences on index fluctuation. The baseline situation is a key factor in both therapeutic decisions and clinical outcomes [51, 61].

Most studies report reproducibility of the same periodontal indices while using different measuring instruments [60, 62, 63], in different clinical situations [31, 33, 59, 60], and evaluating clinical correlation or reproducibility of the same indices [64]. However, only few studies report the daily periodontal indices trend [65].

The results of this present study highlight the clinical effectiveness of cause-related therapy in genders, smokers and nonsmokers, and also additional correlations such as the direct relationship of periodontal indices during the examination phase.

The main aim of this present study is to analyze periodontal indices to ascertain whether or not these indices remain unchanged during the day during the examination phase. In the examination phase, significant changes of FMPS, FMBS, and PRA, but not of PSR, were recorded. Similar significant variations were also observed in nonsmokers. The substantial changes detected in some of the aforementioned periodontal indices have no easy explanation. Periodontal risk assessment variation during the examination phase is significant. However, statistical analysis of PRA changes over time (during the examination phase) showed a statistically similar trend to not only FMBS but also to both FMPS and PSR.

Multivariate analysis can help us to understand these variations by showing that a rise in either PRA-b or PSR-b increases PSR Δ-check (with cumulative effect) and that a rise in either FMBS-b or NoC increases FMBS Δ-checks. Separately, a rise in either PSR-b or female gender increases FMPS Δ-checks, and a rise in either FMBS-b or nicotinism increases PRA Δ-checks (with cumulative effect). Among the considered variables, any one related to either lifestyle or biologic condition of the subject appears to influence Δ-check widths in a different manner. However, many of the measured variables significantly associated with Δ-checks are periodontal indices recorded at baseline and produce an increase of Δ-check widths. Therefore, the worst periodontal indices at baseline produce the widest daily fluctuation at the examination phase, considering the cumulative effect of overall influencing factors. It is probable that, at baseline, subjects suffering from serious periodontal conditions show greater periodontal state instability which is further increased by presence of risk factors such as nicotinism. Nevertheless, patients enrolled in this present study suffered from only mild periodontitis in the worst case. Patients were compliant with study prescriptions and observed the stringent supportive protocol and, on the whole, showed clinically favorable indices during the examination phase.

Biological processes displaying endogenous fluctuation had been widely observed in animals and human beings. It is probable that this fluctuation stimulates physiological mechanisms that promote adjustment to environmental circumstances, favoring systemic requirements [66, 67]. It is hard to separate behavioral and physiological influences on the daily index fluctuation, also considering the mutual correlation between those factors. Different check times during the day may characterize dissimilar situations due to systemic physiology, habits, lifestyle, activities, hygiene, and nutrition. It is known that the alimentary canal macrobiome also shows short-term oscillations related to diet and lifestyle [19–21] and that these oscillations also seem to occur in the oral cavity. However, there are fewer studies and less evidence regarding this [26]. Gingival flow rate and human crevicular fluid flow vary during the day in relation to eating, chewing, and other stimuli [68, 69], particularly in presence of gingival inflammation and the tonicity of saliva increases with saliva flow rate. Additionally, daily oscillations in the expression of inflammatory, immunological, and promigratory molecules have also been described in humans. Several chronic disease symptoms or presentations are known to be exacerbated by daily stressors and worrying which create a stress vulnerability factor [11, 18]. Gene transcription also seems to be time of day dependent in some cases [8]. Even human fibroblasts could have substantial daily variations [70]. Periodontal disease is substantially a set of inflammatory disorders largely supported by microbiota but also supported by other risk and prognostic factors.

The examination phase was performed during three different time slots: early morning, late morning, and early afternoon. These times of day seem to have a neurophysiologic and hormonal meaning in both mammals [71] and humans and are linked to circumstances and different habits related to working rhythms, dietary habits, and lifestyle.

Very few studies comparing the same periodontal indices during the day are found in the literature. In this present study, repeated measurements at different times on the same day were performed in compliant patients affected by mild periodontitis. Repeated measurements performed at different times along several days would introduce greater behavioral and/or physiological effects. This present study observed fluctuations within the same indices on the same day. These fluctuations were significant and significantly proportional with respect to the clinical severity of periodontal damage at baseline but not at examination phase. It is therefore conceivable that these fluctuations are more important in patients affected by severe or serious periodontal disease due to the fact that the pathological substrate may still be active also after stabilization of the clinical condition. It is also possible that daily fluctuations of the oral microbiota, inflammatory and immunological regulation, and even cellular constituents of periodontal tissue amplify indices variation. However, this present study demonstrates that the discrepancy between index measurements seems to be directly related to clinical severity of the periodontal condition at baseline. Therefore, it is conceivable that in patients affected by severe periodontal disease the recognizable variations could become dramatic even if the single index measurement is performed at a second phase after baseline when signs of periodontal damage are much less evident. This could even misdirect the therapeutic strategy due to the fact that the predictive measurement referable to the patients' therapeutic feasibility of recovery could be unknown.

## 5. Conclusion

Our results showed significant variation within indices directly related to clinical severity of the periodontal condition at baseline. Patients affected by severe periodontal disease may show much larger index changes. Since the indices are routinely recorded only once, the index daily variation may have actual clinical significance. This could greatly affect therapeutic strategy because correct periodontal assessment requires multiple evaluations with standardized timing, particularly when the baseline condition is severe.

## Figures and Tables

**Table 1:** Systemic and specific inclusion criteria.

Systemic criteria	Absence of relevant medical conditions (subjects with a clear medical history and no physical or psychological condition, psychotic disorders, personality disorders, which could affect their conduct in the study).
	Smoking status: nonsmokers or smokers up to 20 cigarettes a day (cigar or pipe smokers or people with a story of alcohol and drug abuse were excluded).
	Education: only patients having almost completed at least compulsory education.
	Pregnant, lactating, and underage patients were excluded.

Local criteria	Subjects having more than 16 teeth, not wearing removable partial dentures, not showing oral parafunctions, and not presenting severe skeletal and occlusal abnormalities or substantial oral dysmorphism were enrolled.
	Oral and periodontal conditions: absence of premalignant lesion of the oral cavity.
	Treatment history: subjects who received scaling root planning, or periodontal surgical treatment in the preceding 6 months, or undergoing recent orthodontic or prosthetic therapy were excluded.
	Level of infection: subjects presenting with severe cariogenicity, stomatitis, acute abscesses, or gingival fistulae were excluded.

**Table 2:** Assessed periodontal indices, grouped by gender and smoker status.

	Baseline	Check-I	Check-II	Check-III
*FMPS*				
Nonsmokers	48.7 ± 13.4	19.8 ± 8.9	17.0 ± 6.7	16.3 ± 5.2
Female	49.7 ± 14.4	22.5 ± 9.3	17.5 ± 6.5	16.5 ± 4.3
Male	47.7 ± 12.7	17.3 ± 7.9	17.2 ± 7.0	16.1 ± 6.0
Smokers	34.7 ± 15.5	20.6 ± 9.0	18.3 ± 7.9	20.4 ± 7.8
Female	32.8 ± 15.5	18.3 ± 8.7	17.8 ± 9.0	22.3 ± 8.6
Male	36.7 ± 15.9	23.1 ± 9.1	18.9 ± 7.0	18.4 ± 6.7
Overall	42.9 ± 15.8	20.1 ± 8.9	17.7 ± 7.1	18.0 ± 6.6
*FMBS*				
Nonsmokers	24.2 ± 11.1	9.9 ± 6.4	9.7 ± 6.2	8.9 ± 6.6
Female	25.3 ± 12.2	10.0 ± 5.6	10.2 ± 5.5	9.7 ± 6.7
Male	23.1 ± 10.2	9.9 ± 7.2	9.3 ± 7.0	8.2 ± 6.7
Smokers	18.5 ± 12.1	8.3 ± 5.5	7.0 ± 6.6	6.5 ± 4.9
Female	17.6 ± 12.7	7.7 ± 6.1	8.2 ± 8.4	7.6 ± 5.8
Male	19.4 ± 12.1	9.0 ± 5.1	5.7 ± 3.6	5.3 ± 3.5
Overall	21.8 ± 11.8	9.2 ± 6.0	8.6 ± 6.4	7.9 ± 6.0
*PSR*				
Nonsmokers	1.63 ± 0.46	0.89 ± 0.26	0.89 ± 0.22	0.86 ± 0.23
Female	1.66 ± 0.49	0.99 ± 0.11	0.97 ± 0.13	0.94 ± 0.12
Male	1.61 ± 0.45	0.79 ± 0.31	0.81 ± 0.26	0.79 ± 0.29
Smokers	1.40 ± 0.46	0.96 ± 0.14	0.86 ± 0.22	0.92 ± 0.15
Female	1.30 ± 0.41	0.96 ± 0.15	0.92 ± 0.18	0.97 ± 0.14
Male	1.51 ± 0.51	0.95 ± 0.13	0.80 ± 0.25	0.86 ± 0.15
Overall	1.54 ± 0.47	0.91 ± 0.22	0.88 ± 0.21	0.89 ± 0.20
*PRA*				
Nonsmokers	16.8 ± 13.9	10.4 ± 10.3	10.4 ± 10.2	9.7 ± 9.7
Female	19.3 ± 17.5	12.0 ± 13.1	12.2 ± 12.9	11.4 ± 11.8
Male	14.4 ± 9.5	8.9 ± 6.8	8.6 ± 6.5	8.1 ± 7.2
Smokers	31.2 ± 16.2	17.6 ± 6.4	15.9 ± 8.2	14.2 ± 5.2
Female	25.5 ± 12.1	16.4 ± 7.7	16.2 ± 10.0	14.8 ± 5.3
Male	37.4 ± 18.4	18.9 ± 4.8	15.5 ± 6.1	13.5 ± 5.2
Overall	22.7 ± 16.4	13.4 ± 9.5	12.6 ± 9.7	11.5 ± 8.4

Values are expressed as mean ± standard deviation. FMPS: full-mouth plaque score; FMBS: full-mouth bleeding score; PSR: periodontal screening and recording; PRA: periodontal risk assessment.

**Table 3:** Nonparametric analysis of dental indices.

	Females		Males		Nonsmokers		Smokers		Overall	
	I	II	I	II	I	II	I	II	I	II
FMPS										
I	—	—	—	—	—	—	—	—	—	—
II	−2.7	—	n.s.	—	n.s.	—	n.s.	—	−2.7	—
III	n.s.	n.s.	n.s.	n.s.	−2.5	n.s.	n.s.	n.s.	−2.1	n.s.
FMBS										
I	—	—	—	—	—	—	—	—	—	—
II	n.s.	—	−2.7	—	n.s.	—	n.s.	—	n.s.	—
III	n.s.	n.s.	−3.3	n.s.	n.s.	−2.5	−2.5	n.s.	−3.0	−2.3
PSR										
I	—	—	—	—	—	—	—	—	—	—
II	n.s.	—	n.s.	—	n.s.	—	−2.2	—	n.s.	—
III	n.s.	n.s.	n.s.	n.s.	n.s.	n.s.	n.s.	n.s.	n.s.	n.s.
PRA										
I	—	—	—	—	—	—	—	—	—	—
II	n.s.	—	−2.2	—	n.s.	—	−2.0	—	n.s.	—
III	n.s.	n.s.	−3.4	−2.2	−2.0	−2.5	−3.0	n.s.	−3.4	−2.4

Significant coefficients after Mann–Whitney test. FMPS: full-mouth plaque score; FMBS: full-mouth bleeding score; PSR: periodontal screening and recording; PRA: periodontal risk assessment. I: check-I; II: check-II; III: check-III; n.s.: not significant.

**Table 4:** Significance of dental indices at examination phase after Spearman test.

	PRA-III	PSR-III	FMBS-III	FMPS-III	PRA-II	PSR-II	FMBS-II	FMPS-II	PRA-I	PSR-I	FMBS-I
FMPS-I	n.s.	n.s.	0.4	0.4	n.s.	0.4	0.5	0.7	n.s.	0.7	0.6
FMBS-I	0.4	n.s.	0.8	n.s.	0.4	0.3	0.9	0.3	0.4	0.4	
PSR-I	n.s.	0.5	0.4	0.4	n.s.	0.6	0.3	n.s.	n.s.		
PRA-I	0.9	n.s.	0.3	0.3	0.9	n.s.	0.4	n.s.			
FMPS-II	n.s.	0.2	n.s.	0.5	n.s.	0.3	n.s.				
FMBS-II	0.4	n.s.	0.9	n.s.	0.4	0.4					
PSR-II	n.s.	0.7	0.4	n.s.	n.s.						
PRA-II	0.9	n.s.	0.4	n.s.							
FMPS-III	0.3	0.4	n.s.								
FMBS-III	0.4	0.3									
PSR-III	n.s.										

Significant coefficients after Spearman test. Only variables abutting almost one significant correlation are reported. FMPS: full-mouth plaque score; FMBS: full-mouth bleeding score; PSR: periodontal screening and recording; PRA: periodontal risk assessment. I: check-I; II: check-II; III: check-III; n.s.: not significant.

**Table 5:** Baseline and maximum gap between check-I, check-II, and check-III of indices after seemingly unrelated regression (SUR) analysis.

	FMPS		FMBS		PSR		PRA	
	b	Δ	b	Δ	b	Δ	b	Δ
Age		−0.13		−0.05				
		(0.021)		(0.011)				
Female						−0.19		
						(0.001)		
NoC						−0.02		
						(0.001)		
FMPS-b	—			−0.07	0.02			
				(0.001)	(0.001)			
FMBS-b			—	0.16	0.03	−0.01	0.7	0.08
				(0.001)	(0.001)	(0.001)	(0.001)	(0.005)
PSR-b	28.2	5.45	14.3		—		−9.1	
	(0.001)	(0.002)	(0.001)				(0.005)	
PRA-b			0.5		−0.01	0.01	—	
			(0.001)		(0.001)	(0.039)		
Δ-NoT	−7.0		−10.8		0.45		14.9	
	(0.007)		(0.001)		(0.001)		(0.001)	

Estimated coefficients and *p* values (in brackets) for the 4 seemingly unrelated regressions. Only variables abutting almost one significant correlation are reported. FMPS: full-mouth plaque score; FMBS: full-mouth bleeding score; PSR: periodontal screening and recording; PRA: periodontal risk assessment; NoC: number of cigarettes (per day); Δ-NoT: number of hopeless teeth extracted during cause-related therapy. b: baseline; Δ: Δ-check (maximum gap between check-I, check-II, and check-III).
